# Charge‐Promoted Self‐Metalation of Porphyrins on an Oxide Surface

**DOI:** 10.1002/anie.202015187

**Published:** 2021-01-14

**Authors:** Larissa Egger, Michael Hollerer, Christian S. Kern, Hannes Herrmann, Philipp Hurdax, Anja Haags, Xiaosheng Yang, Alexander Gottwald, Mathias Richter, Serguei Soubatch, F. Stefan Tautz, Georg Koller, Peter Puschnig, Michael G. Ramsey, Martin Sterrer

**Affiliations:** ^1^ Institute of Physics NAWI Graz University of Graz Universitätsplatz 5 8010 Graz Austria; ^2^ Peter Grünberg Institute (PGI-3) Forschungszentrum Jülich 52425 Jülich Germany; ^3^ Jülich Aachen Research Alliance (JARA) Fundamentals of Future Information Technology 52425 Jülich Germany; ^4^ Experimentalphysik IV A RWTH Aachen University 52074 Aachen Germany; ^5^ Physikalisch-Technische Bundesanstalt (PTB) 10587 Berlin Germany

**Keywords:** charge transfer, interfaces, metalation, porphyrins, thin films

## Abstract

Metalation and self‐metalation reactions of porphyrins on oxide surfaces have recently gained interest. The mechanism of porphyrin self‐metalation on oxides is, however, far from being understood. Herein, we show by a combination of results obtained with scanning tunneling microscopy, photoemission spectroscopy, and DFT computations, that the self‐metalation of 2H‐tetraphenylporphyrin on the surface of ultrathin MgO(001) films is promoted by charge transfer. By tuning the work function of the MgO(001)/Ag(001) substrate, we are able to control the charge and the metalation state of the porphyrin molecules on the surface.

## Graphical abstract

The self‐metalation of 2*H*‐tetraphenylporphyrin on the surface of MgO is believed to be feasible only at defect sites. It is shown that this reaction is promoted by charge transfer and does not necessarily require the presence of defects. Using ultrathin MgO films as substrate and tuning its work function allows the charge and metalation state of the adsorbed molecules to be controlled.

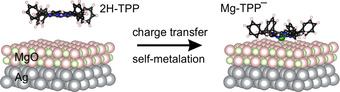

Due to their versatility, porphyrins and metalloporphyrins have attained major attention in all aspects of organic–inorganic hybrid materials science. In particular, surface‐mediated processes of and with porphyrins have strongly contributed to the unbroken fascination of these materials.^[1]^ Surface science investigations of porphyrin self‐assembly, on‐surface synthesis of 2D covalently linked metal–organic frameworks and building of 3D heteroarchitectures,^[2]^ or studies on directed metalation^[3]^ and ligation to control their magnetic, sensing, and catalytic properties, have provided fundamental insight into their chemical and physical properties.^[4]^

The surface‐confined porphyrin metalation is typically achieved by pre‐ or post‐deposition of metal adatoms, or by self‐metalation, which occurs on specific metal substrates such as Cu, Ni, and Fe.^[3a]^ These redox‐type reactions, which include conformational intermediates, hydrogen‐transfer processes and, finally, H_2_ release, are reasonably well understood.^[5]^ Recently, self‐metalation reactions have also been observed on several single‐crystalline oxide surfaces, including TiO_2_[110],^[6]^ Co_*x*_O_*y*_ thin films,^[7]^ and MgO thin films.^[8]^ In contrast to the metalation with metal adatoms, self‐metalation on oxides can be viewed as an ion exchange process, where the two protons of a free‐base porphyrin are replaced by a metal cation. Detailed mechanistic aspects of these reactions remain, however, still elusive. Herein, we provide compelling evidence that the self‐metalation of 2*H*‐tetraphenylporphyrin (2H‐TPP) is promoted by charge transfer on the surface of ultrathin MgO(001) films. Furthermore, by controlling the support properties, we show that uncharged/unmetalated and charged/metalated molecules can be deliberately formed.

The protons released from the free‐base porphyrin during the self‐metalation process on oxides are suggested to contribute to hydroxylation of the surface.[^8^, ^9^] In the case of the self‐metalation of 2H‐TPP on bulk MgO the hydroxy formation indeed provides a substantial energy contribution that makes the metalation reaction thermodynamically feasible.^[8]^ However, this process is strongly morphology‐dependent and studies on MgO nanocubes show that the reaction is limited to low‐coordinated sites such as edges and corners, where the Mg^2+^ vacancy formation energy is lower and hydroxy groups are more stable than on regular surface sites.[^8^, ^9b^] In contrast to this finding is the observation of self‐metalation of a complete 2H‐TPP monolayer on MgO(001) thin films.^[9a]^ While MgO thin film samples also exhibit a certain concentration of surface defects,^[10]^ their limited abundance can, however, not explain the high degree of metalation occurring on the regular surface of the films, which remains a mystery. This calls for a different mechanism of the self‐metalation reaction on the thin film surface that requires detailed knowledge and understanding of the morphology and electronic properties of the combined molecule–substrate system to be unraveled. Herein, we tackle this problem by a combination of experiments using scanning tunneling microscopy (STM) and photoemission spectroscopy, and density functional theory (DFT) computations (Supporting Information SI.1, SI.2).

Large‐scale STM images of the thin MgO(001) film before and after 2H‐TPP adsorption are shown in Figure 1 a,b. As we discuss later, our X‐ray photoelectron spectroscopy (XPS) results confirm the spontaneous metalation of a monolayer of 2H‐TPP on the films, such that it can be assumed that in the STM images most of the porphyrin molecules are already in the metalated, Mg‐TPP, state. The molecules are arranged in a highly ordered square phase with two rotational domains. This is also evident from the low energy electron diffraction (LEED) pattern shown in the Supporting Information (SI.1), which can straightforwardly be interpreted in terms of a $\left(\begin{array}{cc} 4 & -2\\ 2 & 4 \end{array}\right)$ superstructure with a square unit mesh with a unit vector of 13.3 Å. More detailed STM images of isolated molecules and of the monolayer phase are presented in Figure 2 a,b. The isolated molecules appeared as a 4‐lobe structure with a depression in the center (Figure 2 a). The four lobes are associated with the phenyl groups and the axes connecting opposing phenyl groups point in the [110] directions. The identification of the individual molecules is less straightforward in the case of the monolayer phase but is aided by the appearance of a molecular vacancy, as in Figure 2 b. This reveals that the circularly arranged 4‐lobe structure does not correspond to the four phenyl groups of a single molecule, but to the phenyls of 4 neighboring molecules, as depicted in Figure 2 b. The STM of the monolayer phase is in perfect agreement with the LEED structure and leads to the schematic surface model presented in Figure 2 c. This arrangement clearly maximizes T‐type interaction between phenyl rings of neighboring molecules.^[11]^ The 4‐lobe appearance of the TPP molecules in STM is in accordance with a tilting and twisting of the phenyl groups of TPP in the adsorbed state due to the strong interaction of the macrocycle with the surface (Figure 2 d).^[12]^ The upward‐tilted phenyl groups dominate the image contrast and, thus, prevent the observation of the frontier orbital structure, which is mostly localized at the macrocycle. However, this information would be required to gain information about the electronic structure of the adsorbed TPP molecules and their charge state. Therefore, we now turn to the more detailed investigation of the occupied electronic states using angular‐resolved ultraviolet photoemission spectroscopy (ARUPS) and XPS.

**Figure 1:**
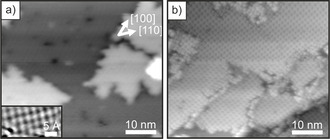
STM images of a) 2 ML MgO(001)/Ag(001) (*U*_bias_=3.0 V, *i*_t_=29 pA), b) 1 ML 2H‐TPP adsorbed on 2ML MgO(001)/Ag(001) at room temperature and annealed at 473 K (*U*_bias_=2.0 V, *i*_t_=28 pA). Inset in (a): atomically resolved image of MgO(001).

**Figure 2:**
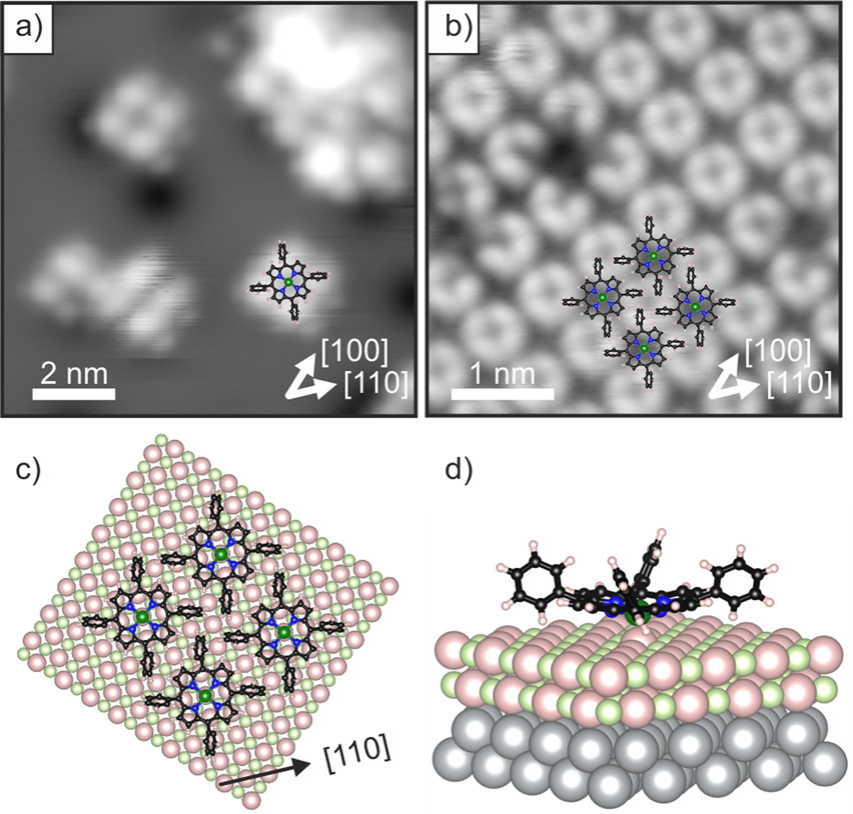
STM images of a) a low‐coverage 2H‐TPP film adsorbed at 80 K on 2 ML MgO(001)/Ag(001) (*U*_bias_=1.2 V, *i*_t_=25 pA) and b) its ordered monolayer (*U*_bias_=3.2 V, *i*_t_=55 pA). c) Tentative structural model of the monolayer phase derived from STM and LEED (top view). d) DFT‐derived adsorption model of Mg‐TPP on 2 ML MgO(001)/Ag(001) (3D view). In this model calculation charge transfer occurs from the substrate into the molecule.

The ARUPS and N 1s XP spectra of clean MgO(001)/Ag(001) and increasing doses of 2H‐TPP on MgO(001)/Ag(001) are presented in Figure 3. Considering the XP spectra first, we observe a single N 1s peak at 399 eV binding energy (BE) upon adsorption of a monolayer 2H‐TPP, suggesting a similar chemical environment of the four N atoms as in metalated TPP.^[13]^ This peak remains upon increasing the 2H‐TPP dose beyond a monolayer, while a pair of additional N 1s peaks at 400.5 and 398 eV, corresponding to the aminic and iminic nitrogen pairs in unmetalated 2H‐TPP,^[13]^ grows in intensity upon further 2H‐TPP adsorption. This confirms the results of previous investigations of the same system and indicates that the 2H‐TPP molecules in the monolayer are metalated to Mg‐TPP, while the molecules in the second and all subsequent layers remain unmetalated 2H‐TPP.^[9a]^ At this point, we note again that the large concentration of metalated TPP on the thin MgO films together with the rather flat terrace‐like morphology of the films (Figure 1) is not consistent with a defect‐mediated self‐metalation reaction suggested in the literature (Supporting Information, SI.3).[^8^, ^9b^] Thus, another driving force for the metalation must be present on the thin MgO films.

**Figure 3:**
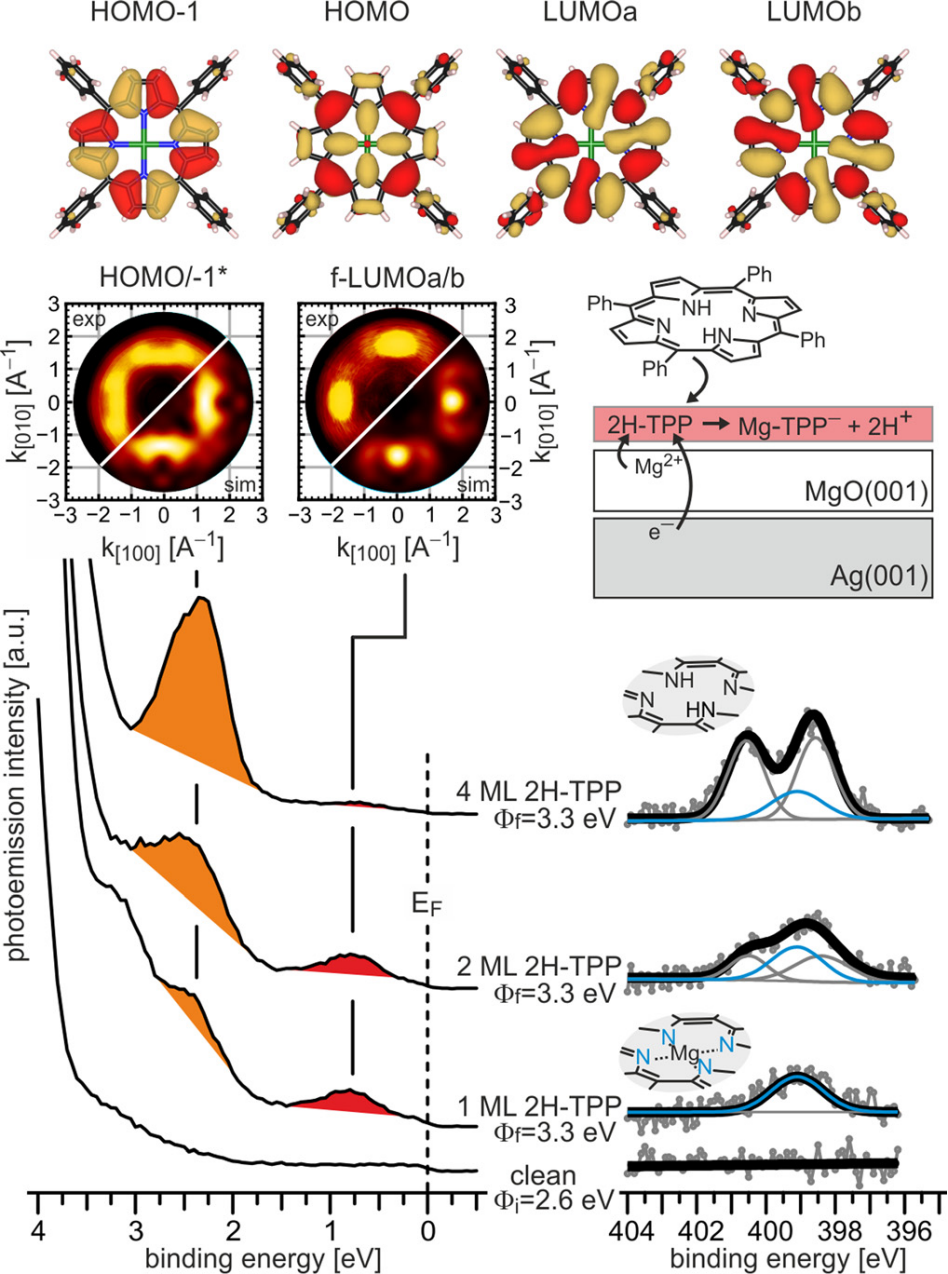
Top: real‐space representation of the HOMO−1, HOMO, and the degenerate LUMOa and LUMOb of gas‐phase Mg‐TPP. ARUPS (bottom, left) and XPS (bottom, right) spectra of clean 2 ML MgO(001)/Ag(001) and increasing doses of 2H‐TPP (1, 2, and 4 ML) at room temperature. Here, *Φ*_i_ is the work function of clean MgO(001)/Ag(001) and *Φ*_f_ is the work function after adsorption of 2H‐TPP. Middle, left: comparison of experimental and simulated momentum maps of 1ML 2H‐TPP on MgO(001)/Ag(001). The experimental maps were taken at the peak maxima of the PE peaks at 0.75 eV, showing the pattern of the superposition of the molecular LUMOa/b, and at 2.3 eV, showing the pattern of the superposition of the HOMO and HOMO−1. Middle, right: schematic model of charging and metalation upon adsorption of 2H‐TPP on MgO(001)/Ag(001).

It is well established that the deposition of MgO films on Ag(001) significantly reduces the Ag(001) work function, which leads to promotion of charge transfer into adsorbates with sufficiently high electron affinity (EA) through electron tunneling.^[14]^ 2H‐TPP has a similar EA (1.6–1.8 eV)^[15]^ to pentacene, which has been shown to become negatively charged on the MgO(001)/Ag(001) substrate.^[16]^ Proof for the charge transfer into the TPP molecules in the present experiments comes from the ARUPS results presented in Figure 3, left. The deposition of 2H‐TPP on 2 ML MgO(001)/Ag(001) thin films, which had an initial work function of *Φ*_i_=2.6 eV, leads to new states in the MgO band gap region that can be associated with the frontier orbitals of 2H‐TPP. Two molecular emissions with BEs of 0.75 and 2.3 eV are immediately present after adsorption of a 2H‐TPP monolayer at room temperature. Simultaneously, the work function increased to 3.3 eV, which is a first indication that charge transfer into the TPP molecules has occurred.^[17]^ Upon increasing the 2H‐TPP coverage to 2 and 4 ML, the 0.75 eV emission is attenuated, while the intensity of the 2.3 eV emission increases with 2H‐TPP coverage, proving that the former is solely due to molecular species in the first monolayer. The additional 2H‐TPP layers do not lead to further work function changes, which indicates that the charge transfer is restricted to the interfacial TPP monolayer.

Identification of the molecular orbitals from which the photoemitted electrons arise is possible with a technique known as photoemission tomography, where the angular intensity distribution of the photoemitted electrons is recorded and converted into a momentum map, which, approximately, corresponds to the reciprocal space image of the real‐space electron density distribution.^[18]^ For the two emissions observed in the MgO band gap region, the experimental momentum maps obtained at BEs corresponding to the peak maxima are displayed in Figure 3 along with simulated maps of the degenerate TPP LUMOs (for the 0.75 eV BE emission) and of the superposition of the HOMO and HOMO−1 (for the 2.3 eV BE emission). See Figure 3, top, for the real‐space representations of the orbitals. Note that the HOMO and HOMO−1 are too close in energy to be resolved in the present experiments. Because of the perfect agreement, the peak at 0.75 eV BE can be identified as emission from an occupied state that has the electron density distribution of the molecular LUMO. This unambiguously confirms the charge transfer into the 2H‐TPP molecules upon adsorption and the corresponding state will henceforth be termed former LUMO (f‐LUMO). Furthermore, since charge transfer through the thin MgO film is accomplished by tunneling, this state is an integer charge transfer state, in agreement with previous observations.[^16^, ^17^]

From the combined XPS/ARUPS data in Figure 3 we conclude that two processes simultaneously take place upon adsorption of 2H‐TPP on ultrathin MgO(001) films: (i) integer charge transfer into the molecules via electron tunneling and (ii) a self‐metalation reaction to Mg‐TPP. Whether these processes are independent of each other, or if one process is the precondition for the other to occur, cannot be answered with the information provided by the experimental data of Figure 3 alone. To prove the interplay between charge transfer and metalation, we block the charge transfer utilizing the recipe developed recently.^[17]^ It relies on chemical modification of the MgO/Ag interface by introducing either oxygen or magnesium. With this, the work function can be tuned over a wide range, from 2.3 to 4.4 eV (Supporting Information SI.3). When charging occurs, the saturation sample work function reached after adsorption of the molecules is equivalent to the critical work function that marks the transition from charging to non‐charging (Supporting Information SI.4).^[17]^ From the experiment shown in Figure 3 the critical work function is at around *Φ*_crit_=3.3 eV. Thus, we expect charge transfer into adsorbed 2H‐TPP to be blocked when adsorbed on an MgO(001)/Ag(001) substrate with *Φ*>3.3 eV.

In Figure 4 a,b we compare the photoemission spectra of 2H‐TPP monolayers adsorbed on MgO(001)/Ag(001) with different initial work function, 2.6 and 3.9 eV, i.e., below and above *Φ*_crit_ for charging. The spectra for 2H‐TPP on the low work function substrate resemble the results reported in Figure 3, where both charge transfer and self‐metalation occur, as shown by the observation of the occupied former LUMO in ARUPS and a single N 1s component in the XPS spectrum. If 2H‐TPP is instead adsorbed on the high work function substrate, the band‐gap state in the ARUPS is absent, confirming that the molecules are not charged. In addition, we observe the typical signature of non‐metalated 2H‐TPP in the corresponding XP spectrum.

**Figure 4:**
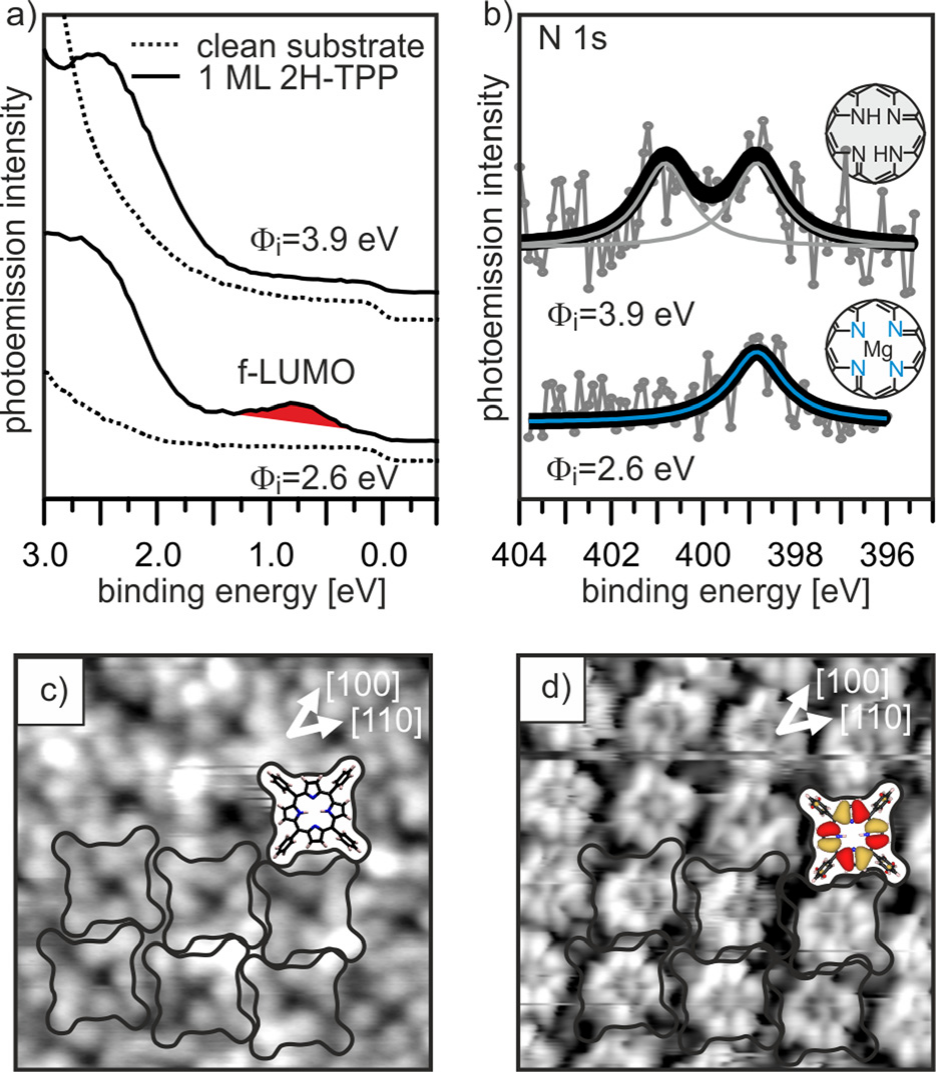
a,b) ARUPS (a) and N 1s XPS (b) for 2H‐TPP adsorbed on 2 ML MgO(001)/Ag(001) films with different initial work function, *Φ*_i_. c,d) STM of 2H‐TPP on high work function MgO(001)/Ag(001) at *U*_bias_=+2.9 V (c) and *U*_bias_=−2.6 V (d).

The absence of charge transfer also has consequences for the appearance of the molecules in STM. Instead of the phenyl‐dominated 4‐lobe appearance as in Figure 2, at similar tunneling conditions both the peripheral phenyls and the pyrrole groups of the macrocycle are imaged (Figure 4 c). Furthermore, at negative tunneling voltage, submolecular contrast resembling the electron density distribution of the HOMO−1 was achieved (Figure 4 d). In addition, we find that, compared to the arrangement shown in Figure 2, the 2H‐TPP molecules are rotated by 45°, i.e., the phenyl axis is now aligned with the [100] crystallographic direction. The different STM contrast suggests that the 2H‐TPP molecules are flatter, and the strong tilting and twisting of the phenyl groups, as seen for the charged and metalated TPP, is absent. This implies a weaker interaction of uncharged 2H‐TPP with the surface compared to the charged and metalated molecule, which also affects the stability of the monolayer phases. While the charged and metalated monolayer is stable up to at least 473 K (see Figure 1 b), a similar thermal treatment led to the structural disintegration of the uncharged and unmetalated 2H‐TPP monolayer.

We could thus show that self‐metalation of 2H‐TPP on ultrathin MgO films is promoted by integer charge transfer from the MgO/Ag interface into the 2H‐TPP LUMO. Most remarkably, this process is not restricted to defects such as low‐coordinated sites at edges and corners but also occurs on defect‐free terrace sites. Previous computational studies revealed that the self‐metalation reaction is energetically unfavored on the terraces of bulk‐like MgO.^[8]^ There, the energy gain due to the formation of hydroxy groups next to the Mg^2+^ vacancy is too small to compensate the high vacancy formation energy. It is obviously the charging of the 2H‐TPP molecules on the ultrathin MgO films that provides the energetic balance to make the metalation reaction on terrace sites thermodynamically more favorable. This can be rationalized in terms of the stronger interaction energy and the reduced surface‐to‐molecule distance for the charged 2H‐TPP molecules. Moreover, the presence of a charged molecule on top of the MgO surface leads to significant rumpling of the surface ions, which could further aid the self‐metalation reaction by lowering the vacancy formation energy.

We note that our findings also provide an explanation for the somewhat contradicting results of previous 2H‐TPP monolayer adsorption studies on MgO thin films, where 50 % metalation has been reported on 10 ML thin MgO films,^[8]^ whereas complete (100 %) metalation was observed on 2 ML thin MgO films.^[9a]^ The reason for this can be found in the electrostatics of the system. The charging is a consequence of potential equilibration in the combined adsorbate–substrate system, which, for the present case, can be explained with a simple capacitor model.^[17]^ In this model, an increase of the dielectric thickness necessitates a decrease of transferred charge to keep a constant potential. Thus, on thicker MgO films less molecules in the monolayer get charged and, consequently, less molecules become metalated (Supporting Information SI.4). It has to be noted that, while our DFT simulations succeeded in describing the charging of 2H‐TPP molecules and the associated work function changes on MgO(001)/Ag(001), the results for the related self‐metalation reaction were not in good agreement with the experimental findings, even with inclusion of various van der Waals correction schemes (Supporting Information SI.5). In general, the metalation reaction of 2H‐TPP on the MgO(001)/Ag(001) surface is a complicated process to simulate, since it bridges ionic binding, weak physisorption and metal–organic charge transfer, all situations that can be well described by ab initio methods individually, but in combination impose a serious challenge and an open task for further computational investigations.

In summary, we have unraveled the mystery of the high 2H‐TPP self‐metalation activity on ultrathin MgO(001)/Ag(001) films by showing that the metalation is promoted by charge transfer. This finding provides important hints for the mechanism of the self‐metalation of porphyrins on oxide surfaces. The charge‐induced conformational changes in the molecule, the decrease of the molecule–substrate distance, and the enhanced surface rumpling may all be relevant parameters that positively influence the reaction pathway. Remarkably, our results suggest a method to control electric and chemical (charged/metalated vs. uncharged/unmetalated) states of porphyrins by tuning the work function of the substrate or thickness of the dielectric, which opens the way for a selective surface functionalization.

Raw data are available at the Jülich DATA public repository.^[19]^

## Conflict of interest

The authors declare no conflict of interest.

## Supporting information

As a service to our authors and readers, this journal provides supporting information supplied by the authors. Such materials are peer reviewed and may be re‐organized for online delivery, but are not copy‐edited or typeset. Technical support issues arising from supporting information (other than missing files) should be addressed to the authors.

Supplementary
