# Supplementary material for: Charge‐Promoted Self‐Metalation of Porphyrins on an Oxide Surface
Source: Angew Chem Int Ed Engl. 2021 Jan 14;60(10):5078–82. doi: 10.1002/anie.202015187 (PMC7986846; doi:10.1002/anie.202015187)
Supplement: Supplementary file 1 — Supplementary [file ANIE-60-5078-s001.pdf]

Angewandte Chemie
Eine Zeitschrift der Gesellschaft Deutscher Chemiker

Supporting Information

Charge-Promoted Self-Metalation of Porphyrins on an Oxide Surface

*Larissa Egger[+], Michael Hollerer[+], Christian S. Kern, Hannes Herrmann, Philipp Hurdax, Anja Haags, Xiaosheng Yang, Alexander Gottwald, Mathias Richter, Serguei Soubatch, F. Stefan Tautz, Georg Koller, Peter Puschnig, Michael G. Ramsey, and Martin Sterrer**

anie_202015187_sm_miscellaneous_information.pdf

Supporting Information

SI.1 Experimental details

All experiments were performed under ultrahigh vacuum conditions in three separate preparation and analysis systems. All chambers were equipped with the tools necessary for sample cleaning/preparation (electron beam heating, sputter gun, metal and molecule evaporators, gas dosing) and basic characterization (low energy electron diffraction, quadrupole mass spectrometer).

Sample preparation

The Ag(001) substrate was cleaned by repeated cycles of sputtering (Ar^+ ions at 0.8-1 kV) and annealing (773 K, 2 min). MgO(001) films were grown by Mg evaporation from an e-beam evaporator in an oxygen environment. Mg fluxes used were of the order of 1 Å/min as monitored by a quartz microbalance. The MgO deposition was done at a substrate temperature of 553 K and in an O_2 pressure of 10^{-6} mbar, followed by slow cooling (roughly 2.5 °C/min), the accepted procedure providing epitaxial MgO(100) films with high structural quality.[1]

2H-TPP was deposited onto the MgO(001)/Ag(001) substrate either at room temperature or 80 K from a Knudsen type molecular evaporator heated to 433K. The molecular flux was calibrated using a quartz microbalance. The low energy electron diffraction (LEED) pattern of a full monolayer of 2H-TPP adsorbed on MgO(001)/Ag(001) thin films shows a $\begin{pmatrix} 4 & -2 \\ 2 & 4 \end{pmatrix}$ superstructure with a square unit mesh with a unit vector of 13.3 Å.

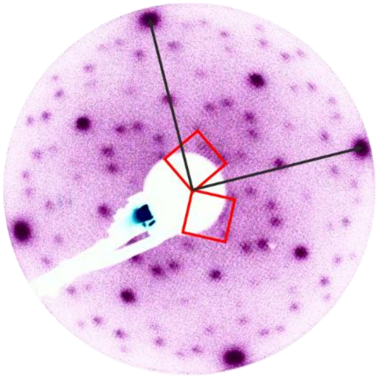

LEED image of the 2H-TPP monolayer on MgO(001)/Ag(001).

Scanning tunneling microscopy (STM)

STM measurements were performed at 77 K with a Createc low-temperature STM attached to an ultrahigh-vacuum preparation chamber (base pressure 2×10^{-10} mbar) using electrochemically etched tungsten tips. The bias was applied to the sample.

Photoemission experiments

Photoemission experiments were performed at the Metrology Light Source of the Physikalisch-Technische Bundesanstalt (Berlin, Germany) at the insertion device beamline using a toroidal electron-energy analyzer. A photon energy of 35 eV and an incidence angle of $\chi = 40°$ with respect to the surface normal were used. The polarization direction is in the specular plane, which is also the measured photoelectron trajectory. The emitted electrons were simultaneously recorded with polar angles of -80° to 80° with respect to the surface normal. For the shown momentum maps, the photoelectron intensity on the positive polar angle range was used. Note that this direction corresponds to the maximal molecular emission relative to the substrate emission. To measure the momentum maps for a chosen binding energy, the sample was rotated in the azimuthal direction in 1° steps, which results in a full photoelectron distribution in the (k_x, k_y) plane perpendicular to the sample normal. The energy distribution curves were obtained by integration of photoemission intensity over the entire available $k_{//}$ range.

Coverage-dependent UPS(He I) and XPS(Mg K_α) experiments were taken in-house with a hemispherical Scienta SES-200 analyser in angular and spatial mode, respectively. ARUPS spectra were taken along the [100] azimuthal direction at $\theta = 40°$ take-off angle. XP spectra were taken at normal emission.

SI.2 Computational details

DFT calculations for the gas-phase orbitals of both 2H-TPP and Mg-TPP have been performed with the ab-initio quantum chemistry software NWChem.[2] We have used a 6-31G* basis set and the GGA-PBE approximation for exchange-correlation effects.[3] Theoretical momentum maps of the angular photoelectron distribution have been simulated within the plane wave final state approximation, from the Fourier transform of the real space orbitals of the isolated molecule, as described in Ref. [4].

In order to shed light on the metalation of 2H-TPP, we have additionally simulated the adsorption of 2H-TPP and Mg-TPP on the MgO/Ag-surface in various configurations by means of DFT utilizing the VASP code and employing the projector-augmented wave method.[5] Using a repeated slab approach, with 5 Ag-layers, 2 MgO layers and one molecule per unit cell, an inter-layer vacuum layer of 13 Å thickness and a dipole-correction in z-direction, all investigated structures have been optimized using a van der Waals corrected GGA functional for exchange and correlation effects.[3, 6] All atomic positions except those of the lowest 3 layers of Ag have been relaxed, and we have used the experimental lattice parameter of Ag with a=4.076 Å. Subsequent to the geometry optimization, the electronic structure has been calculated using an energy cutoff of 400 eV, a Gaussian-type smearing with a broadening of 0.2 eV and a Brillouin zone-sampling with 8x8x1 points centered around the Gamma point.

SI.3 Work function tuning and influence of defects

A typical MgO thin film preparation results in samples with an average work function of 2.8 +/-0.2 eV, which is similar to the calculated work function for an MgO(001)/Ag(001) system with ideal, i.e. stoichiometric, interface (see below and [7]). Based on previous experimental reports[8] and theoretical predictions[7b] we applied various treatments to alter the work function. To reduce the WF, we typically mildly annealed the samples after preparation in UHV or evaporated additional Mg during annealing. To increase the WF, the samples were exposed to oxygen (5×10^{-7} to 2×10^{-4} mbar) at elevated temperature after preparation. For the experiments presented in this paper, the MgO films were either used as obtained from a typical preparation (WF = 2.7 eV), or additionally exposed to oxygen to increase the WF (WF = 3.9 eV). As shown below (Computational results, SI5), the work function variation can be accomplished by inserting additional oxygen atoms in the interstitial sites of the topmost Ag layer (WF increase), or be replacing Ag atoms in the topmost Ag layer by Mg (WF reduction). Neither of these treatments changes the stoichiometry of the MgO film on top of the Ag(001) substrate. (We mention that it is also possible to change the work function by introducing either oxygen or magnesium vacancies in the interfacial MgO layer,[9] but this is experimentally less likely at the applied conditions.)

Of course, some defects can and will be formed during the MgO film growth and several previous studies have reported on defect characterization on MgO thin films. The most obvious surface defects on MgO thin films are low coordinated sites at step edges.[10] Additionally, color centers (oxygen vacancies) have attracted a lot of interest in the past.[11] They can either exist with 0, 1, or 2 electrons trapped inside the vacancy. In particular, the F^+ and F^0 centers (1 and 2 trapped electrons, respectively) lie energetically above the MgO valence band and, therefore, can influence the reactivity of the MgO surface. There are, however, no indications for a large and countable abundance of color centers on freshly prepared MgO thin film samples, neither from scanning tunneling microscopy,[12] electron spin resonance,[13] UPS and metastable impact electron spectroscopy,[10b,14] and electron energy loss spectroscopy[15] studies. It is known that color centers can be formed on thin MgO films by relatively harsh measures such as electron bombardment.[13,15]
It is, therefore, safe to say that for the results presented in this manuscript, defects do not play a significant role for the observed charge transfer into 2H-TPP on the ultrathin MgO films. This is supported by both the initial work function dependence and the MgO layer thickness dependence of charging and metalation, which follows the expectations of the capacitor model and relies only on charge transfer from the MgO-Ag interface to the molecules on the surface. If surface defects would be responsible for the charge transfer into the 2H-TPP molecules, the capacitor model would not be valid anymore, which is not observed (see SI4).

SI.4 Results of additional experiments

Adsorption of Mg-TPP on high work function MgO(001)/Ag(001)

To provide an additional proof that the state at 0.75 eV binding energy is due to charge transfer into the LUMO of 2H-TPP, and is not related to an intrinsic electronic state of Mg-TPP, which is formed by self-metalation on the MgO surface, we have deposited Mg-TPP on a high WF 2 ML MgO(001)/Ag(001) substrate. In Figure S1 we compare the UPS spectra of this preparation with the UPS spectra of 2H-TPP deposited on low WF and high WF 2 ML MgO(001)/Ag(001). As reported

in the main manuscript, only on low WF MgO(001)/Ag(001) the state at 0.75 eV is present, according to charge transfer from the substrate into the molecular LUMO. The deposition of 1 ML Mg-TPP on a high WF substrate, where no charge transfer occurs, gives rise to an UPS spectrum without any new features directly below the Fermi energy. The broad peak at 2 eV BE is related to the HOMO and HOMO-1 emissions of neutral Mg-TPP.

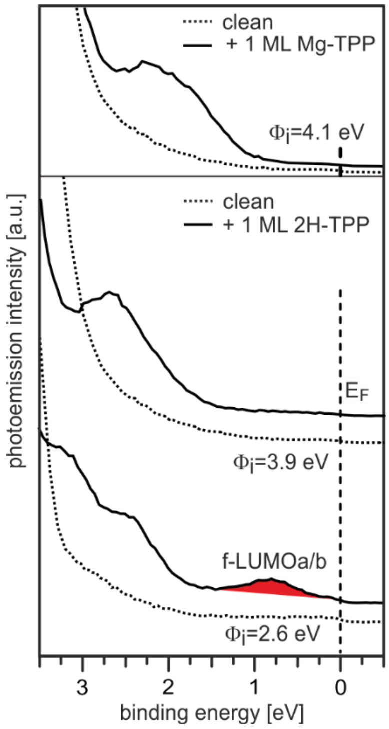

Figure S1. Comparison of UPS spectra after adsorption of a monolayer 2H-TPP on 2 ML MgO(001)/Ag(001) films with different initial work function (bottom) and for a monolayer Mg-TPP on a high work function 2 ML MgO(001)/Ag(001) film.

Work function and MgO film thickness dependence of charging and self-metalation

According to our previous findings,[9,16] if the work function is low enough for charge transfer to occur into adsorbates on MgO(001)/Ag(001) thin films (Fermi level pinning regime), the number of charged molecules on the surface is related to the potential difference ΔΦ (work function difference before and after adsorption) according to:

$$\Delta\Phi = \frac{\sigma d_{cs}}{\varepsilon_0 \varepsilon_r} \quad \text{(capacitor model, Eq. S1)}$$

where σ is the average charge density in the molecular film [C/m²], ε_r is the dielectric constant of the thin film and d_{cs} (charge separation distance) is the distance between the charge at the molecule and the interface. Thus, for the same MgO film thickness (same d_{CS}), the number of charged molecules depends on the initial work function and decreases as Φ_i increases.

We have tested this hypothesis by studying the charge transfer into and self-metalation of 2H-TPP on 2 ML MgO(001)/Ag(001) preparations with different initial work function. As shown in Figure S2, the experimental results are in agreement with the expectations from the capacitor model: as the initial work function is increased, the intensity of the state at 0.75 eV BE in UPS (Figure S2, left), which is related to the occupied former LUMO of the TPP molecules, decreases until it is no longer present on samples with high initial work function, where no charge transfer takes place. Concomitant with

the decrease of the former LUMO intensity in UPS, the N 1s XPS peak related to metalated TPP (Mg-TPP) decreases and the fraction of unmetalated 2H-TPP increases (Figure S2, right). Note that for an initial work function of 3.3 eV, which is the same as the observed pinning work function that marks, in an ideal system, the transition from charging to non-charging, there is still a large fraction of charged and metalated TPP present on the surface. This result can be understood based on the creation of an additional dipole due to a push-back effect of the adsorbed TPP molecules, which partly counterbalances the charge-induced dipole. The "true" pinning work function is therefore a few tenths of an eV above the observed pinning work function (see also the discussion in Ref. [9]). From the relative intensities of the N 1s XP signals the fraction of charged/metalated and uncharged/unmetalated TPP molecules in the monolayer can be calculated. For the $\Phi_i = 3.3$ eV work function sample shown in Figure S2, around 30% of the molecules in the monolayer are charged/metalated and 70% of the molecules are uncharged/unmetalated. This is also consistent with the f-LUMO intensity seen in the ARUPS.

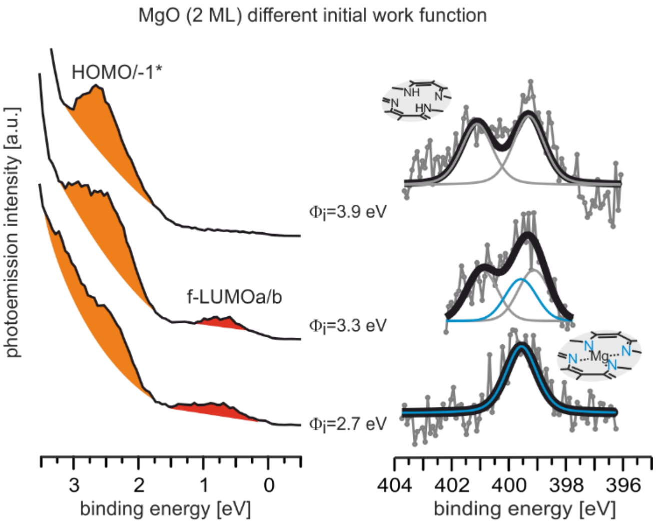

Figure S2. ARUPS (left) and N 1s XPS (right) spectra of 2H-TPP adsorbed on 2ML MgO(001)/Ag(001) samples with different initial work function (Φ_i). Increasing initial work function reduces charge transfer (f-LUMO) and metalation.

Next, we have studied the MgO film thickness dependence of the charging and self-metalation process. According to Eq. S1, the number of charged molecules should decrease at constant potential difference $\Delta\Phi$ if the MgO film thickness (and thus d_{CS}) is increased.[9] We have prepared MgO films with the same initial work function ($\Phi_i = 2.7$ eV) but different thickness (nominally 2 ML, 4 ML, and 8 ML) and show in Figure S3 the UPS (left) and N 1s XPS (right) results for the adsorption of a monolayer 2H-TPP on these samples. For the 2 ML and 4 ML thin MgO(001) films the results are almost identical and suggest that only charged and metalated TPP molecules are present on the surface. However, we observe a decrease of the occupied former LUMO peak in UPS and, as a result, the co-presence of metalated (50%) and non-metalated TPP (50%) molecules according to XPS, for the 8 ML thin MgO film, in perfect agreement with the expectations from the capacitor model (Eq. S1).
The additional results presented here thus provide further proof for the direct relation between charge transfer into and self-metalation of 2H-TPP on the surface of ultrathin MgO(001)/Ag(001) films.

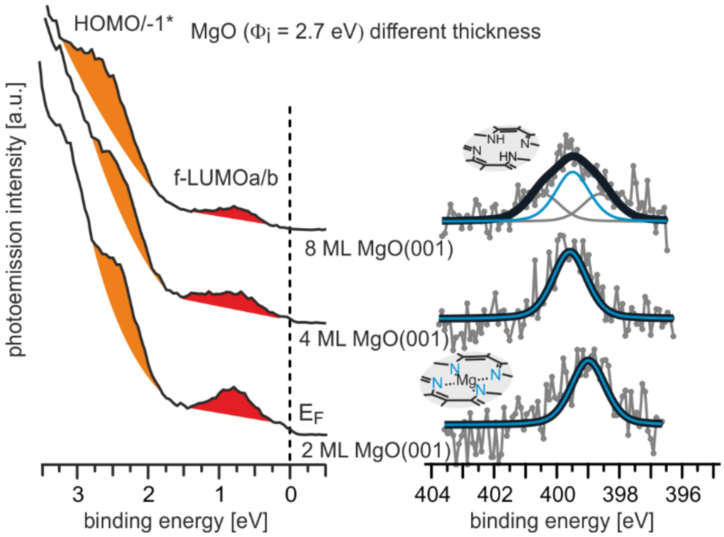

Figure S3. ARUPS (left) and N 1s XPS (right) spectra of 2H-TPP adsorbed on MgO(001)/Ag(001) samples with different MgO film thickness. Increasing MgO thickness (with the same initial work function) reduces charge transfer (f-LUMO) and metalation (XPS).

SI.5 Computational results

An overview over the simulated structures can be seen in Figure S4. Importantly, we have also investigated the influence of the substrate's work function (WF) on the electronic structure of the adsorbed molecule. The DFT calculation for two stoichiometric layers of MgO on five layers of Ag results in an initial WF of 3.0 eV prior to the adsorption of the molecule (Figure S4, middle row). When incorporating an additional 1/4 monolayer of oxygen atoms at interstitial sites in the topmost Ag-layer, the WF rises to 4.7 eV (Figure S4, top row) as has already been described in Ref. [7b]. Conversely, substituting 1/4 of the Ag atoms in the topmost Ag layer by Mg lowers the initial WF to 2.1 eV (Figure S4, bottom row).[8] Using these three model substrates, both the adsorption of 2H-TPP (Figure S4, middle column) and Mg-TPP (Figure S4, right column) has been simulated. Note that for the adsorption of Mg-TPP, the metalation reaction has been simulated as an exchange reaction consisting of the removal of a Mg^{2+} from the topmost MgO layer and the concomitant creation of a hydroxyl group in the so-created Mg vacancy. As can be seen from Figure S4, the preferred adsorption site of 2H-TPP is the center of the molecule being on top of an oxygen ion (red balls) with the nitrogen atoms sitting on top of Mg^{2+} (orange balls) resulting in an azimuthal orientation of the molecule in agreement with experimental findings. When studying the exchange reaction for the metalation process we have tested several relative positions of the hydroxyl groups (protons highlighted as green balls) with respect to the molecule's central Mg^{2+} ion (highlighted as dark blue ball). It turns out that in the most favorable adsorption configuration, Mg-TPP's center is again situated on top of an oxygen atom and the hydroxl groups are located in the topmost MgO layer at the position of the Mg^{2+} vacancy underneath a nitrogen atom of Mg-TPP.

The resulting electronic structures have been analyzed in Figure S4 in terms of their total density of states (DOS, black lines) and the DOS projected onto the molecule (red lines). In all cases one can

clearly identify the energetic positions of the frontier molecular orbitals as indicated in the figure. While for the high WF case no charge transfer into the LUMO takes place, neither for the 2H-TPP nor the Mg-TPP case, the situation is markedly different for the initial WF of 3.0 eV. Here the calculation results in the formerly doubly degenerate LUMO peak to be centered at the Fermi energy, clearly indicating 1e charge transfer into the LUMO. It should be noted that the energy of the LUMO peak truncated by E_F must be taken as an artifact of the computational treatment. As has been shown for other molecular systems adsorbed on thin insulating layers on metals integer charge transfer leads to the singularly occupied molecular orbital (SOMO) appearing well below E_F and the singularly unoccupied molecular orbital (SOMO) well above it.[16,17] The creation of supercells containing more than one molecule and the usage of a hybrid functional with a substantial fraction of exact exchange[17-18] would be necessary to realize conditions that allow for integer charge transfer. Unfortunately, in the present case, such a theoretical treatment is prevented by the large number of atoms per unit cell owing to computational reasons. Nevertheless, from our experience,[17] we expect that the electronic structure shown for the initial WF of 3.0 eV would lead to an integer charge transfer corresponding to the experimentally observed situation of the f-LUMO (SOMO) 0.8 eV below E_F. Finally, when reducing the initial WF to 2.1 eV by incorporating additional Mg atoms in the topmost Ag layer, the LUMO peak moves almost entirely below the Fermi energy thereby further promoting the charge transfer into the molecule with the LUMO fully occupied (2e).

In order to tackle the question of the relative energetic stability of the adsorption of intact 2H-TPP on MgO(001)/Ag(001) compared to the metalated Mg-TPP on MgO(001)/Ag(001) as resulting from the exchange reaction described above, we now analyze the adsorption energies resulting from our DFT total energy calculations. We define the adsorption energy as follows:

$$E_{ads} = E_{tot} - (E_{surf} + E_{mol}) \qquad \text{(S2)}$$

Here, E_{tot} refers either to the total energy of 2H-TPP/MgO(001)/Ag(001) or the Mg-TPP/MgO(001)/Ag(001) complex subsequent to the metalation reaction, and E_{surf} and E_{mol}, respectively, refer to the total energies of the uncovered MgO(001)/Ag(001) substrate prior to the adsorption and the gas-phase 2H-TPP molecule's total energy. With this definition of the adsorption energy, negative values correspond to bonding to the surface while positive values suggest that adsorption is not favored.

To investigate the role that van-der-Waals (vdW) corrections play for the adsorption energies and to demonstrate possible complications inherently present in state-of-the-art vdW-corrected DFT functionals when applied to mixed metallic/ionic/molecular hybrid systems, we have evaluated the adsorption energies for four different functionals. First, we disregard vdW corrections entirely, that is, we employ the standard PBE-GGA functional (GGA).[3] Second, we use Grimme's empirical D3 method,[6] which we have already used for the electronic structure analysis discussed above. In addition, we have employed two more vdW-correction schemes, namely the SCAN+rVV10 method (SCAN)[19] and the Tkatchenko-Scheffler (TS)[20] method with parameters obtained from the self-consistent screening equation. It should be noted that for each functional all structures have been relaxed prior to the evaluation of Eq. S2.

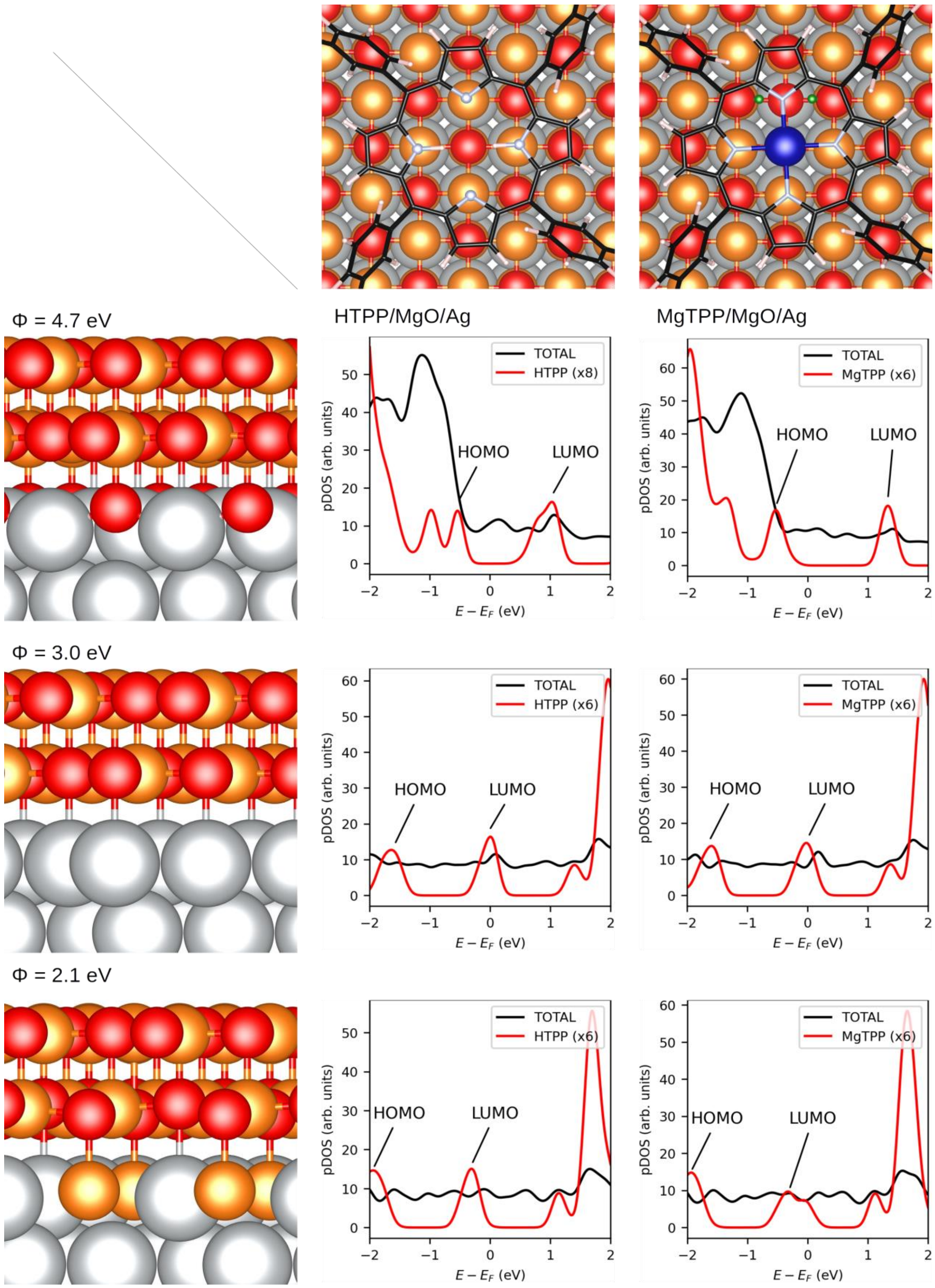

Overview of total DOS (black) and DOS projected on molecule (red) for 3 different initial WF (rows) for 2H-TPP and Mg-TPP on MgO(001)/Ag(001) (columns). In the real space models the red, orange and grey balls represent O, Mg and Ag atoms, respectively, while the small green balls represent the displaced hydrogen.

The resulting adsorption energies as well as the final work function values are listed in Table S1. First, we note that in a pure GGA calculation without vdW correction, neither 2H-TPP nor the metalated Mg-TPP would bind at all, or at least only very lightly, on the high and medium WF substrates. This finding is in agreement with earlier GGA calculations for Mg-TPP on bulk MgO cubes,[21] where the self-metalation reaction was found to be thermodynamically favorable at step edges of MgO but not on a defect-free MgO surface. Only for the very low initial WF does PBE predict binding, which can be explained by the substantial electron transfer. Importantly, when comparing the situation before and after the metalation, we observe that PBE predicts the metalation reaction to be favored except for the low WF substrate where PBE yields almost equal adsorption energies for the two molecules.

Table S1. Adsorption energies according to Eq. S2 and final work functions for 2H-TPP and Mg-TPP for different initial WFs for four the different DFT functionals GGA, D3, SCAN and TS, respectively. See text for more details.

Initial WF (eV)	Adsorbate	Adsorption energy (eV)				Final WF (eV)			
		GGA	D3	SCAN	TS	GGA	D3	SCAN	TS
4.7	2H-TPP	0.42	-3.41	-5.01	-7.10	4.52	4.16	3.96	4.17
4.7	Mg-TPP	-0.08	-3.61	-5.18	-6.62	4.55	4.43	4.32	4.46
3.0	2H-TPP	0.72	-4.00	-6.22	-7.76	3.17	3.20	3.36	3.19
3.0	Mg-TPP	0.44	-3.70	-5.90	-6.87	3.12	3.16	3.25	3.13
2.1	2H-TPP	-1.84	-6.22	-8.55	-8.43	3.03	3.17	3.24	3.12
2.1	Mg-TPP	-1.72	-5.71	-7.79	-6.42	3.06	3.07	3.11	2.98

It is a well-known fact that vdW interactions, which are not properly accounted for in a GGA functional, play an essential role in the adsorption of organic molecules on all organic and inorganic substrates. When inspecting the adsorption energies obtained for Grimme's empirical DFT+D3 correction, we indeed notice the strong influence of dispersive long-range interactions on the energy balance for the adsorption. First, the adsorption energies now clearly indicate that, both, 2H-TPP and Mg-TPP will stick to the MgO(001)/Ag(001). Secondly, we observe that the binding becomes more favorable with the increased charge transfer into the molecule on decreasing the substrate's initial WF. However, DFT+D3 predicts the metalation reaction to be endothermic except for the high WF substrate. There are several reasons which might explain this theoretical outcome conflicting somewhat with the experimental findings. First, it should be noted that, although we have tested several configurations for the position of the hydroxyl group relative to the Mg-TPP adsorption position, we may have missed the global energy minimum of the metalated Mg-TPP complex on the surface. Secondly, it should be stressed that the present case of an organo-metallic complex adsorbed on an ionic crystal double-layer residing on a metallic substrate challenges common vdW correction schemes. For instance, while pure GGA is known to describe ionic crystals well, it fails to correctly describe weak physisorption of molecules on surfaces.[22] Thus, the known deficiencies of common vdW-correction schemes for ionic bonds may prevent the delicate interplay between bonding of Mg to the MgO-lattice and the Mg-N interaction inside Mg-TPP to be correctly accounted for. We have indeed tested how other vdW-correction schemes perform for the present systems. Summarizing the results listed in Table S1, we find quite a strong effect on the absolute size of the adsorption energies,

e.g., the adsorption energies from the SCAN and TS schemes are significantly larger than those from the D3 approach. However, also the latter approaches would predict the metalation to be less favored. As a third and final reason for this discrepancy, it should be emphasized that the charge transfer into the molecule plays a key role for the metalation. While the GGA+vdW approach captures the overall correct trend as a function of the substrate's WF (compare discussion above and results of Figure S4), it must be noted that a more realistic description of the charge transfer would probably require the utilization of hybrid DFT functionals.[18] Unfortunately, however, a full geometry relaxation using hybrid functionals is presently computationally out of reach for such large unit cells.

In general, the metalation reaction of 2H-TPP on the MgO(001)/Ag(001) surface is a complicated system to simulate, since it bridges ionic binding, weak physisorption and metal-organic charge transfer, all situations that can be well described by ab-initio methods individually, but in combination impose a serious challenge and an open task for further computational investigations and developments.

References:

[1] J. Pal, M. Smerieri, E. Celasco, L. Savio, L. Vattuone, M. Rocca, *Phys. Rev. Lett.* **2014**, *112*, 126102.

[2] M. Valiev, E. J. Bylaska, N. Govind, K. Kowalski, T. P. Straatsma, H. J. J. Van Dam, D. Wang, J. Nieplocha, E. Apra, T. L. Windus, W. de Jong, *Comp. Phys. Commun.* **2010**, *181*, 1477-1489.

[3] J. P. Perdew, K. Burke, M. Ernzerhof, *Phys. Rev. Lett.* **1996**, *77*, 3865-3868.

[4] P. Puschnig, S. Berkebile, A. J. Fleming, G. Koller, K. Emtsev, T. Seyller, J. D. Riley, C. Ambrosch-Draxl, F. P. Netzer, M. G. Ramsey, *Science* **2009**, *326*, 702-706.

[5] a) G. Kresse, J. Furthmüller, *Phys. Rev. B* **1996**, *54*, 11169-11186; b) G. Kresse, D. Joubert, *Phys. Rev. B* **1999**, *59*, 1758-1775.

[6] S. Grimme, J. Antony, S. Ehrlich, H. Krieg, *J. Chem. Phys.* **2010**, *132*, 154104.

[7] a) S. Ling, M. B. Watkins, A. L. Shluger, *Phys. Chem. Chem. Phys.* **2013**, *15*, 19615-19624; b) J. Pal, M. Smerieri, E. Celasco, L. Savio, L. Vattuone, R. Ferrando, S. Tosoni, L. Giordano, G. Pacchioni, M. Rocca, *J. Phys. Chem. C* **2014**, *118*, 26091-26102.

[8] T. Jaouen, P. Aebi, S. Tricot, G. Delhaye, B. Lépine, D. Sébilleau, G. Jézéquel, P. Schieffer, *Phys. Rev. B* **2014**, *90*, 125433.

[9] P. Hurdax, M. Hollerer, P. Puschnig, D. Lüftner, L. Egger, M. G. Ramsey, M. Sterrer, *Adv. Mater. Interfaces* **2020**, *7*, 2000592

[10] a) M. Sterrer, T. Risse, H. J. Freund, *Surface Science* **2005**, *596*, 222-228; b) Y. D. Kim, J. Stultz, D. W. Goodman, *Langmuir* **2002**, *18*, 3999-4004.

[11] G. Pacchioni, *ChemPhysChem* **2003**, *4*, 1041-1047.

[12] M. Sterrer, M. Heyde, M. Novicki, N. Nilius, T. Risse, H. P. Rust, G. Pacchioni, H. J. Freund, *J. Phys. Chem. B* **2006**, *110*, 46-49.

[13] a) M. Sterrer, E. Fischbach, T. Risse, H. J. Freund, *Phys. Rev. Lett.* **2005**, *94*, 4; b) M. Sterrer, E. Fischbach, M. Heyde, N. Nilius, H. P. Rust, T. Risse, H. J. Freund, *J. Phys. Chem. B* **2006**, *110*, 8665-8669.

[14] A. Kolmakov, J. Stultz, D. W. Goodman, *J. Chem. Phys.* **2000**, *113*, 7564-7570.

[15] J. Kramer, W. Ernst, C. Tegenkamp, H. Pfnür, *Surface Science* **2002**, *517*, 87-97.

[16] P. Hurdax, M. Hollerer, L. Egger, G. Koller, X. S. Yang, A. Haags, S. Soubatch, F. S. Tautz, M. Richter, A. Gottwald, P. Puschnig, M. Sterrer, M. G. Ramsey, *Beilstein J. Nanotechnol.* **2020**, *11*, 1492-1503.

[17] M. Hollerer, D. Lüftner, P. Hurdax, T. Ules, S. Soubatch, F. S. Tautz, G. Koller, P. Puschnig, M. Sterrer, M. G. Ramsey, *ACS Nano* **2017**, *11*, 6252-6260.
[18] O. T. Hofmann, P. Rinke, M. Scheffler, G. Heimel, *ACS Nano* **2015**, *9*, 5391-5404.
[19] a) J. Sun, A. Ruzsinszky, J. P. Perdew, *Phys. Rev. Lett.* **2015**, *115*, 036402; b) H. Peng, Z.-H. Yang, J. P. Perdew, J. Sun, *Phys. Rev. X* **2016**, *6*, 041005.
[20] a) A. Tkatchenko, M. Scheffler, *Phys. Rev. Lett.* **2009**, *102*, 073005; b) A. Tkatchenko, R. A. DiStasio, R. Car, M. Scheffler, *Phys. Rev. Lett.* **2012**, *108*, 236402; c) T. Bučko, S. Lebègue, J. Hafner, J. G. Ángyán, *Phys. Rev. B* **2013**, *87*, 064110.
[21] J. Schneider, M. Franke, M. Gurrath, M. Rockert, T. Berger, J. Bernardi, B. Meyer, H. P. Steinrück, O. Lytken, O. Diwald, *Chem. Eur. J.* **2016**, *22*, 1744-1749.
[22] M. Kim, W. J. Kim, T. Gould, E. K. Lee, S. Lebègue, H. Kim, *J. Am. Chem. Soc.* **2020**, *142*, 2346-2354.
